# Supplementary material for: Comprehensive Annotation and Functional Exploration of MicroRNAs in Lettuce
Source: Front Plant Sci. 2021 Dec 24;12:781836. doi: 10.3389/fpls.2021.781836 (PMC8739914; doi:10.3389/fpls.2021.781836)
Supplement: Supplementary file 2 [file Data_Sheet_8.ZIP › GSM2230749.plot/Lsa-miRN55_Lsat_1_v5_gn_7_10221.1_5814_TPlot.pdf]

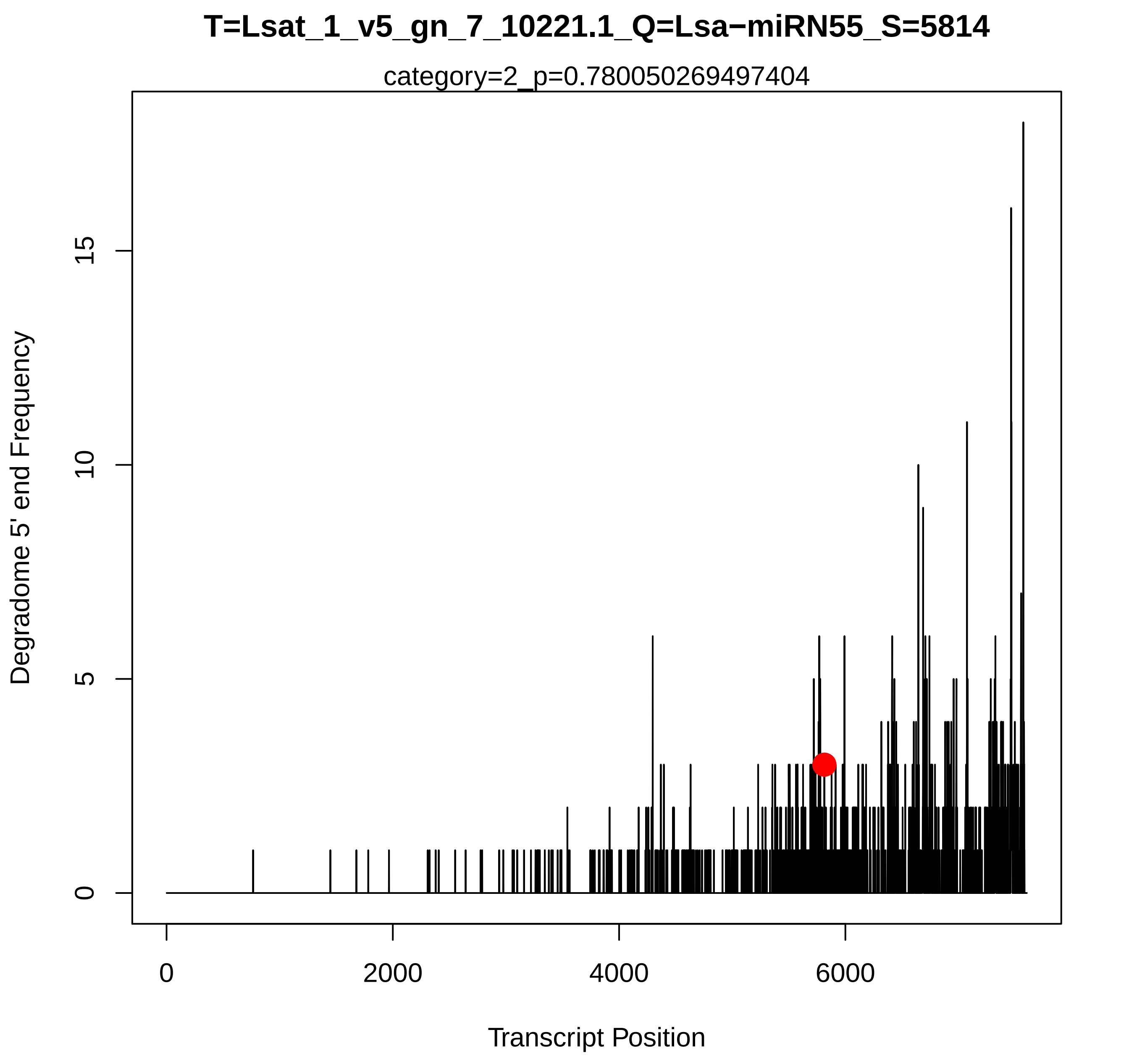

T=Lsat_1_v5_gn_7_10221.1_Q=Lsa−miRN55_S=5814
category=2_p=0.780050269497404
Degradome 5' end Frequency
0
5
10
15
0
2000
4000
6000
Transcript Position
